# The Role of Interface Band Alignment in Epitaxial SrTiO_3_/GaAs Heterojunctions

**DOI:** 10.1021/acsaelm.4c01150

**Published:** 2024-09-16

**Authors:** Shaked Caspi, Maria Baskin, Sergey Shay Shusterman, Di Zhang, Aiping Chen, Doron Cohen-Elias, Noam Sicron, Moti Katz, Eilam Yalon, Nini Pryds, Lior Kornblum

**Affiliations:** † The Andrew & Erna Viterbi Dept. of Electrical and Computer Engineering, Technion−Israel Institute of Technology, Haifa 32000-03, Israel; ‡ The Israel Center for Advanced Photonics, 81800 Yavne, Israel; § Applied Physics Division, Solid State Physics Department, Soreq NRC, 81800 Yavne, Israel; ∥ Center for Integrated Nanotechnologies (CINT) Los Alamos National Laboratory Los Alamos, Los Alamos, New Mexico 87545, United States; ⊥ Department of Energy Conversion and Storage, Technical University of Denmark (DTU), DK-2800 Kongens Lyngby, Denmark

**Keywords:** functional oxides, semiconductors physics, band structure, strontium titanate, gallium arsenide

## Abstract

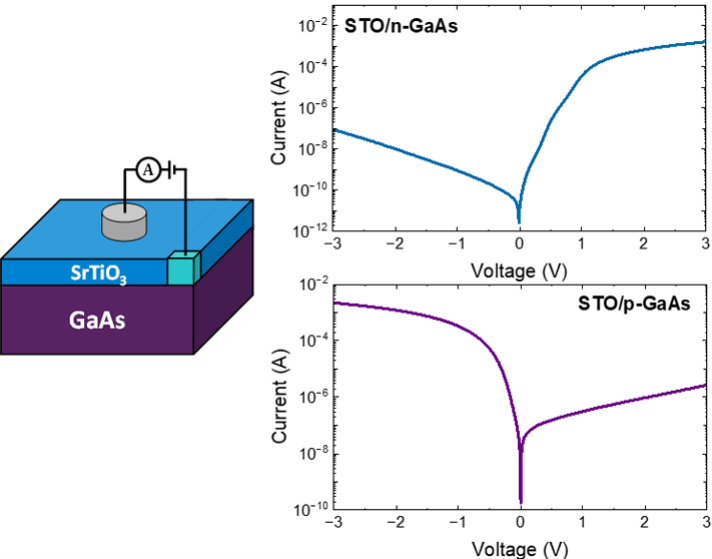

Correlated oxides are known to have remarkable properties, with a range of electronic, magnetic, optoelectronic, and photonic functionalities. A key ingredient in realizing these properties into practical technology is the effective and scalable integration of oxides with conventional semiconductors. Unlocking the full spectrum of functionality requires atomically abrupt oxide–semiconductor interfaces and intimate knowledge of their potential landscape and charge transport. In this study, we investigated the electrical properties of epitaxial SrTiO_3_/GaAs heterostructures by examining the band alignment and transport behavior at the interface. We employ X-ray photoelectron spectroscopy (XPS) to measure the barriers for electrons and holes across the interface and, through them, explain the transport behavior for junctions with n- and p-type GaAs. We further show qualitative evidence of the strong photoresponse of these structures, illustrating the potential of these structures in optoelectronic devices. These results establish the fundamental groundwork for utilizing these interfaces toward new devices and define their design space.

## Introduction

Functional oxides have been at the forefront of condensed matter physics for decades, owing to their rich spectrum of correlated-electron phenomena. Notable examples include high-temperature superconductivity, multiferroicity, metal–insulator transitions, two-dimensional (2D) electron gases, and others.^1^ In addition to the lively fundamental interest in these phenomena, they are hailed for their functionality and attractive prospects in novel electronic, magnetic, and photonic devices.^2−4^ Indeed, significant focus has been given recently to harnessing these properties for crafting functional devices.

Integration of complex oxides with semiconductors has been gaining significant traction as a route to combine the functionality of oxide electronics with conventional microelectronics and optoelectronics technologies.^5^ Since these oxides typically need to be single crystalline to access their functionality, the traditional route for integration with semiconductors has been by epitaxy. While challenging, intensive research in oxide epitaxy on semiconductors led to the development of effective growth techniques on all common semiconductors,^5−8^ using industry-compatible instruments.^5^ In parallel, a recent breakthrough^9^ with epitaxial lift-off of oxide membranes allows the transfer of crystalline layers,^10^ heterostructures,^11^ and twisted stacks^12^ onto many substrate types, including semiconductors.^13,14^ While this approach is attractive, as it circumvents epitaxy on semiconductors, the scalability of epitaxial lift-off to wafer-scale processes remains to be developed.

In addition to the combination of oxide electronics with conventional semiconductor circuits, another level of integration harnesses the coupling of oxides with semiconductors to form new functionalities. These can include, for example, ferroelectric devices^15,16^ and photoelectrocatalytic devices.^17,18^ Achieving these functionalities requires an atomically abrupt oxide–semiconductor interface. Unintended oxide–semiconductor interface layers, which are challenging to prevent, compromise the oxide functionality and inhibit or even block electrical currents across the interface. Molecular beam epitaxy (MBE) remains the unrivaled route for obtaining clean and abrupt oxide–semiconductor interfaces,^5^ with the added benefit of being industry-compatible and scalable.^19^

While significant work has been done on the fundamentals of the interfaces of epitaxial oxides with silicon^6^ and germanium,^8,20^ oxide-GaAs interfaces received less attention, where most of the previous work revolved around the atomic structure.^21−24^ While prior works attempted to probe the electronic transport of oxide-GaAs heterojunctions,^25,26^ atomically abrupt interfaces are crucial for careful elucidation of the electronic structure. Thus, the band structure and transport behavior at the oxide-GaAs interfaces remain largely unexplained despite their critical role in the performance and design space of functional devices.

Here, we employ spectroscopic and electrical analyses to unveil the electronic properties of epitaxial SrTiO_3_/GaAs interfaces. Structural characterization of MBE-grown epitaxial SrTiO_3_ (STO) on GaAs is presented, and the band alignment at the interface is determined by X-ray photoelectron spectroscopy (XPS). The electronic transport behavior across the STO/GaAs heterojunction is analyzed both for n-type and p-type GaAs and explained via the band structure. The results reveal opposite rectifying behavior in these different types of heterostructures, and the source for these different behaviors is examined and discussed.

## Experimental Section

GaAs epitaxial layers were grown using a Veeco GEN-20A solid source molecular beam epitaxy (MBE) system equipped with a Mark-V valved As-cracker and dual filament effusion cells for group-III elements. 2 μm thick doped layers were grown at a rate of 0.88 μm/hour on double-side polished 4″ (100) GaAs semi-insulating wafers (2° off to ⟨110⟩) at 580 °C. Growth temperature was measured using kSA BandiT in Band Edge mode. Be and Si were used for p-doping and n-doping, respectively. The doping level of all the GaAs layers used in this work is p, n = 3 × 10^17^ cm^–3^, as determined by Hall measurements.

In order to prevent GaAs surface oxidation when exposed to ambient conditions, an amorphous arsenic capping layer was deposited by keeping the As-cracker valve open while cooling the wafer down to 50 °C. The arsenic layer thickness of ∼200 nm was determined using X-ray reflectometry.

10 mm × 10 mm pieces were cleaved from the GaAs wafers for the SrTiO_3_ (STO) growth. Oxide epitaxy was performed in a Veeco GenXplor oxide molecular beam epitaxy (MBE) system at a base pressure of ∼1 × 10^–10^ Torr. Fluxes were calibrated in vacuum before each growth using a quartz crystal microbalance (QCM) to a value of 1 unit cells (u.c.) per minute. Molecular oxygen was introduced to the chamber via a needle valve. Upon loading the GaAs substrate to the oxide MBE chamber, the As capping layer was desorbed by heating the substrate to ∼500 °C in ultrahigh vacuum (UHV) conditions.^22,27^ This process was used for exposing the pristine GaAs surface on which the oxide film was then grown, as monitored by reflection high-energy electron diffraction (RHEED).^28^ The RHEED evolution during growth was reported elsewhere.^27^ The temperature was measured by a thermocouple in proximity (but not in contact) to a Mo backplate clamped to the back of the GaAs substrate.

For the epitaxial growth of the STO films, an initial half-monolayer of Ti was deposited at low temperature (340 °C).^29,30^ This was followed by shuttered deposition^31^ of Sr and Ti at a low O_2_ pressure of ∼1 × 10^–7^ Torr, resulting in a few monolayers of amorphous STO. The “Motorola process”^6,32^ was employed, where several cycles of few-monolayer amorphous STO were annealed at ∼650 °C (without oxygen) and then cooled down again for another deposition. This method allows separation of heat from oxygen exposure, which is important for preserving the oxide–semiconductor interface. A typical cycle consists of 3–10 u.c., and all of the final films have a nominal thickness of 30 uc (∼12 nm). Cooling to room temperature at a rate of 30 °C/min was performed without oxygen until 500 °C and with oxygen (5 × 10^–7^ Torr) at lower temperatures.

Structural characterization of the STO films was done using X-ray diffraction (XRD), performed on a Rigaku SmartLab diffractometer. The surface of the oxide films was imaged by atomic force microscopy (AFM) using an Asylum Research MFP-3D Infinity instrument operated in tapping mode. Scanning transmission electron microscopy (STEM) images were taken on an image-corrected FEI Titan 80–300 S/TEM microscope operated at 300 keV. The TEM specimens were prepared by the conventional method, including gluing, grinding, polishing, and ion milling multisteps. X-ray photoelectron spectroscopy (XPS) measurements were acquired with a PHI Versaprobe III instrument using a monochromated Al Kα source (1486.6 eV) and a takeoff angle of 45°. The XPS data were analyzed using CasaXPS software, and the curve fitting of the spectra was performed after Shirley background subtraction. For the analysis of the high-resolution XPS spectra, the binding energies (BE) were calibrated according to the C 1s peak (285.0 eV). For band structure analysis, energies were aligned by setting the Ti 3p peak at 37.6 eV to match the literature.^33^ Two structures were investigated using XPS: a 10 unit cells (∼4 nm) STO film on n-type GaAs (n = 3 × 10^17^cm^–3^) and a 30 u.c. (∼12 nm) STO film on p-type GaAs (p = 3 × 10^17^cm^–3^).

Current–voltage (*I*–*V*) measurements are performed inside a light-sealed probe station using a Keithley 2450 source-meter instrument. Positive voltage is defined as the voltage applied to the top electrodes, prepared by deposition of 50 nm Pt or Al pads, with an area of 1.1 × 10^–4^ cm^2^, on the STO surface. To achieve electrical contact to the doped GaAs layer, a scratch in the STO film was made at the corner of the sample, and a metal electrode of Au/Ti (100:5 nm) was then deposited and annealed at 350 °C for 5 min using rapid thermal annealing (RTA, JIPelec JetFirst 200 HT) under N_2_ atmosphere. Deposition of the metal electrodes was performed using e-beam evaporation through a shadow mask.

## Results and Discussion

**Figure 1:**
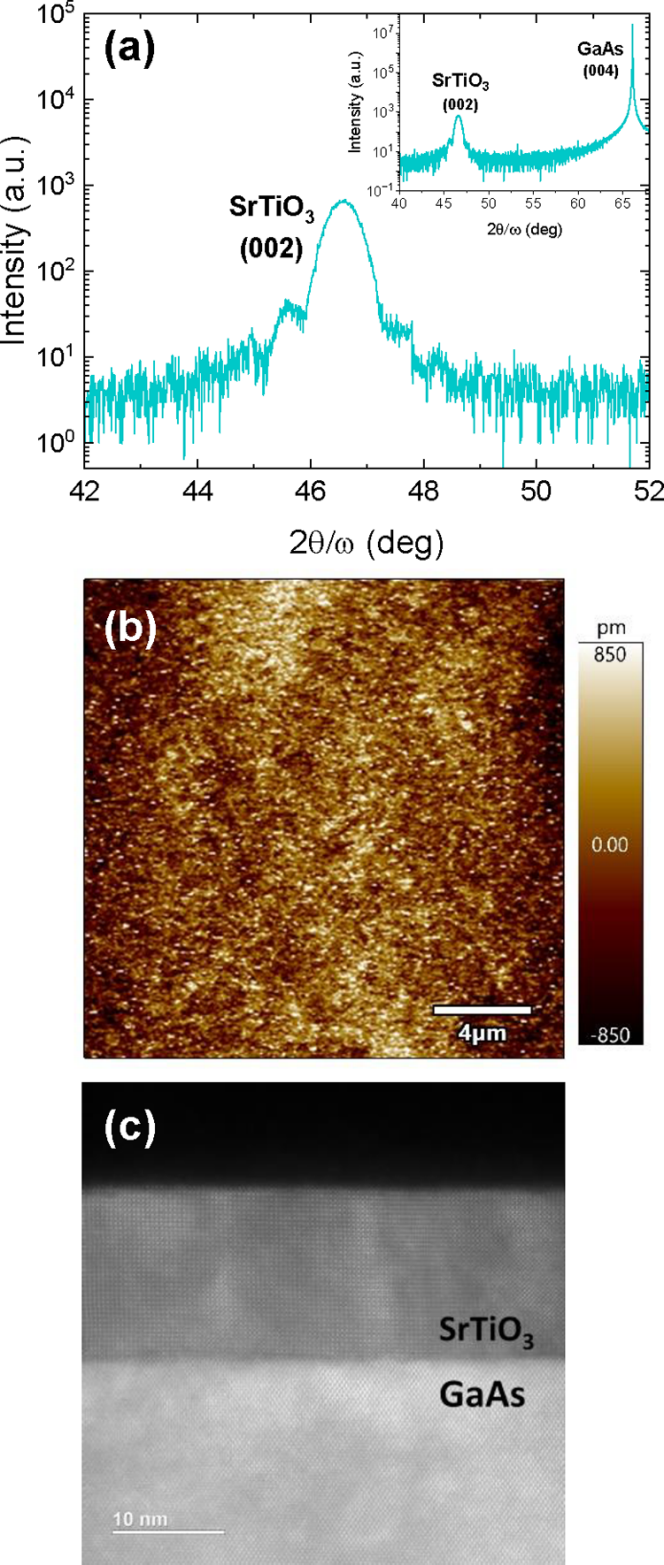
Microstructure of the STO/GaAs heterostructure. (a) X-ray diffraction of the (00l) planes, (b) AFM image, and (c) Cross-sectional HAADF-STEM micrograph of “thick” 12 nm STO on GaAs.

The structural quality of the SrTiO_3_ (STO) films (thickness of 12 nm) grown epitaxially on GaAs substrates is confirmed by X-ray diffraction (XRD). The intense (002) STO peak seen in the diffraction in Figures 1a and S1 indicates the high crystallinity of the film, while no foreign phases are observed. Kiessig fringes shown in Figure 1a indicate high-thickness homogeneity and low roughness. Atomic force microscopy (AFM) imaging (Figure 1b) reveals a smooth surface of the STO films, with root-mean-square (RMS) roughness under 0.9 nm (see also Figure S2 and Table S1). The low roughness of the surface is required for good contact between the STO and the deposited top electrodes.

Cross-sectional scanning transmission electron microscopy (STEM) image (Figure 1c) shows abrupt interfaces, which are important for coupling between the electronic functionalities of the oxide and the GaAs semiconductor.

**Figure 2:**
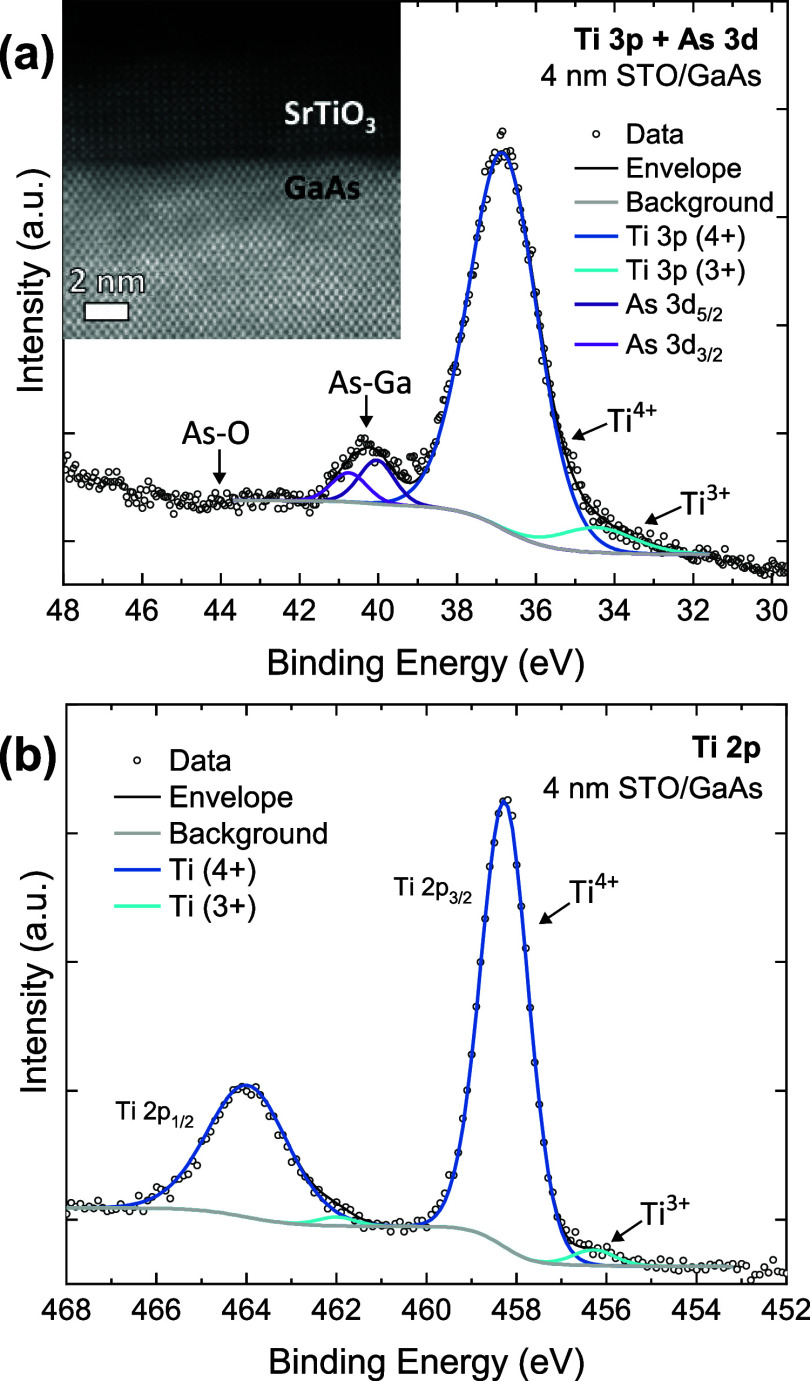
XPS spectra of core levels (a) Ti 3p and As 3d, and (b) Ti 2p, and a cross-sectional HAADF-STEM micrograph (inset) of “thin” 4 nm STO on GaAs.

XPS analysis is performed on two STO/GaAs samples, one with a 10 unit cells (u.c., ∼4 nm) STO film (“thin”) and another with a 30 u.c. (∼12 nm) STO film (“thick”). The thin layer is used to acquire photoelectrons from both STO and n-type GaAs at the interface, whereas the thick sample allows a comparison with the “bulk” of the STO film without contribution from GaAs.^34^ The interfacial chemistry is probed by analyzing the core level spectrum of Ti 3p and As 3d (Figure 2a), acquired from the thin structure in which the sampling depths include both the oxide and the underlying GaAs substrate. The As 3d_5/2_ peak is positioned at a binding energy (BE) of 40.04 eV, assigned to As ions in the GaAs lattice, while no signal is observed at higher BE that are typical of As–O bonds (∼42–45 eV).^35,36^ This verifies the absence of oxidized arsenic species and hence rules out oxidation of the GaAs substrate during the growth of the STO film. This conclusion is consistent with the clean interface seen in the STEM image of the same structure (inset of Figure 2a). The Ti 2p spectrum acquired from the thin sample (Figure 2b) is fitted with a major Ti^4+^ peak at a BE of 458.26 eV and another weak peak at 456.28 eV (fitted with a spin–orbit splitting of 5.71 eV). The lower BE component, with a relative area of 3% of the signal, is assigned to the Ti^3+^ state.^37,38^ The presence of the reduced Ti (Ti^3+^) is attributed to oxygen deficiency in the STO lattice due to the limited oxygen pressure (∼1 × 10^–7^ Torr) in the MBE chamber during growth. This limitation stems from the attempt to avoid oxidation of the semiconductor surface and to preserve the STO/GaAs interface. As a result, the STO film is likely to behave as an n-type semiconductor due to the induced oxygen vacancies that function as electron donors.^39^ For STO grown on GaAs under comparable conditions, in-plane electron mobility values in the range of 0.5–1 cm^2^ V^–1^ s^–1^ were reported.^27^

**Figure 3:**
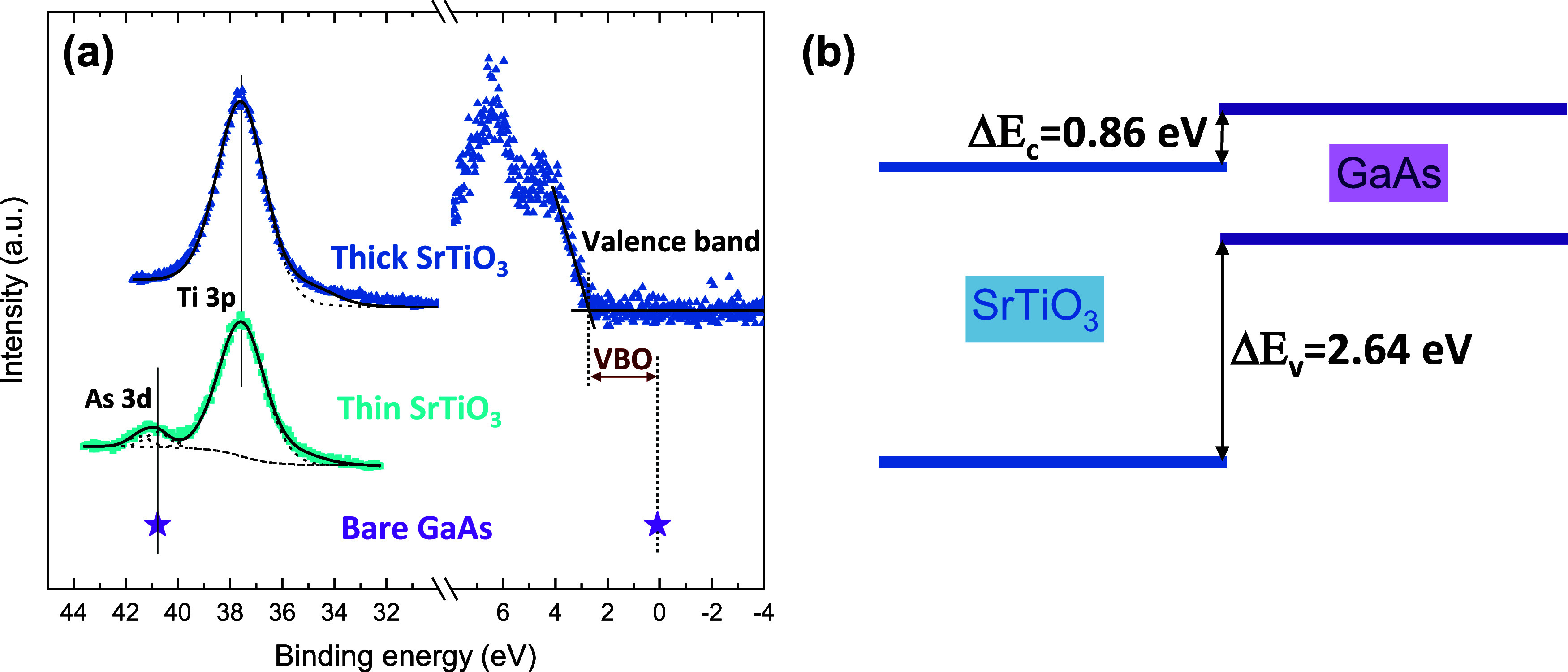
(a) XPS spectra of As 3d and Ti 3p and valence band of structures with thick (top) and thin (bottom) SrTiO_3_ films on GaAs, and data of clean GaAs.^18^ (b) Schematic band diagram with conduction and valence band offsets at the SrTiO_3_/GaAs interface under flat band condition.

Determination of the band offsets at the STO/GaAs interface is performed using the Kraut method^40^ by analyzing the valence band edge (VBE) and the core level XPS spectra of the thick and thin structures^34,41,42^ (Figure 3a, where data for bare GaAs is taken from a previous work,^18^ where a similar arsenic desorption procedure was carried out inside the XPS chamber). The valence band offset (VBO, Δ*E*_v_) is determined by the energy difference between the VBE of the STO, measured for the thick STO structure, and the VBE of bare GaAs. The BE scales of the bare GaAs and the thick structure are aligned with each other through the energetic distance between As 3d and Ti 3p, as measured in the thin structure. This allows the quantification of the relative distance between the valence band edges of STO and GaAs, giving a VBO value of Δ*E*_v_ = (*E*_As 3d_ – *E*_VBE_)_GaAs_ – (*E*_Ti 3p_ – *E*_VBE_)_thick STO_ – (*E*_As 3d_ – *E*_Ti 3p_)_thin STO_ = 2.64 ± 0.15 eV. With the band gap values of GaAs (1.42 eV) and STO (3.2 eV), the conduction band offset (CBO, Δ*E*_c_) is determined to be Δ*E*_c_ = 0.86 ± 0.15 eV, in agreement with previous reports.^18,43^Figure 3a depicts a graphic equivalent of this method, where the Ti 3p peak is aligned to 37.6 eV^33^ and the other spectra are aligned accordingly (as indicated by the vertical lines). This provides the calibrated VBE difference. A schematic energy diagram is constructed, as presented in Figure 3b, assuming flat band condition (omitting possible band bending inside the semiconductor^8,20,44,45^). Since we compare the core levels of the oxide and semiconductor, the band offset results include all of the possible surface contributions.

The properties of the Type II (staggered) band alignment that is formed at the epitaxial STO/GaAs interface (Figure 3b) directly affect the carrier transport across the heterojunction. Electrons are expected to readily flow from the GaAs to the oxide, while the large VBO forms a substantial potential barrier for the transport of holes in that direction. We note that the entire analysis does not account for the possibility of internal electric fields, thereby assuming a flat band condition. The ability to fit the Ti 2p features with a large 4+ peak (97% of the total area, Figure 2b) indicates that such fields are negligible inside the STO.^46^ While some band bending is likely in GaAs, we sample the very top of the semiconductor, a consequence of the shallow information depth under the STO, and therefore, the band bending is not expected to affect the offset calculation. We provide an uncertainty range of 0.15 eV for the band offset results, which is much larger than the fitting uncertainty, in acknowledgment of the small, unlikely effect of doping on the band offset. We note that recent advances in synchrotron XPS analysis allow the construction of the full band diagram, including band bending,^7,8,44,45,47^ which is a worthy cause for future work.

**Figure 4:**
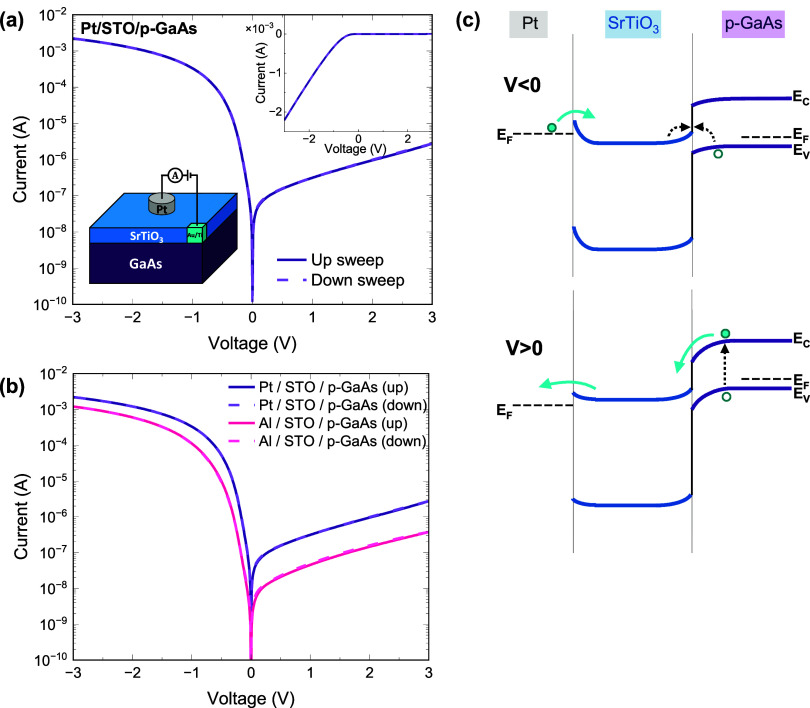
(a) Current–voltage curves of the Pt/STO/p-GaAs structure in semilogarithmic and linear (top-right inset) scales, and a schematic diagram of STO/GaAs device structure (bottom-left inset). (b) Comparison between Pt and Al top electrodes. (c) Schematic energy band diagram of Pt/STO/p-GaAs structure under positive and negative voltages. Note that the typical band bending in GaAs is much wider than the thickness of the STO layer.

Vertical current–voltage (*I*–*V*) analysis was employed to understand the transport behavior across the STO/GaAs interface. Two types of heterostructures were analyzed, where 12 nm thick STO films were grown on p-type GaAs (p-structure) and n-type GaAs (n-structure). The schematic illustration presented in the inset of Figure 4a demonstrates the structure of the measured devices. The *I*–*V* measurements were conducted between a Pt pad on top of the STO and a Ti/Au contact to the GaAs substrate; the voltage is defined as positive on the Pt while the GaAs is grounded, as depicted in the illustration.

The p-structure exhibits distinct rectifying behavior (Figure 4a) with a rectification ratio of 10^3^ at ±1 V. Under low forward bias, the dependence between current and applied voltage can be described by , where *I*_0_ is the reverse saturation current, *q* is the electron charge, *n* is the ideality factor, *k* is the Boltzmann constant, and *T* is the temperature.^48^ An ideality factor of 1.8 is determined from the slope of the semilogarithmic *I*–*V* characteristics (Figure 4a) at a voltage range between −0.2 and −0.06 V, by . The large VBO at the STO/GaAs junction forms a potential barrier that limits the transport of holes from the semiconductor to the STO side. Hence, the forward current is governed by electrons that recombine with holes that are injected from the p-GaAs side (Figure 4c). Being closer to 2 rather than to 1, the ideality factor implies a considerable contribution of recombination processes that take place inside the depletion region in the p-GaAs side and at the STO/p-GaAs interface, with the latter being more predominant.^48−50^

**Figure 5:**
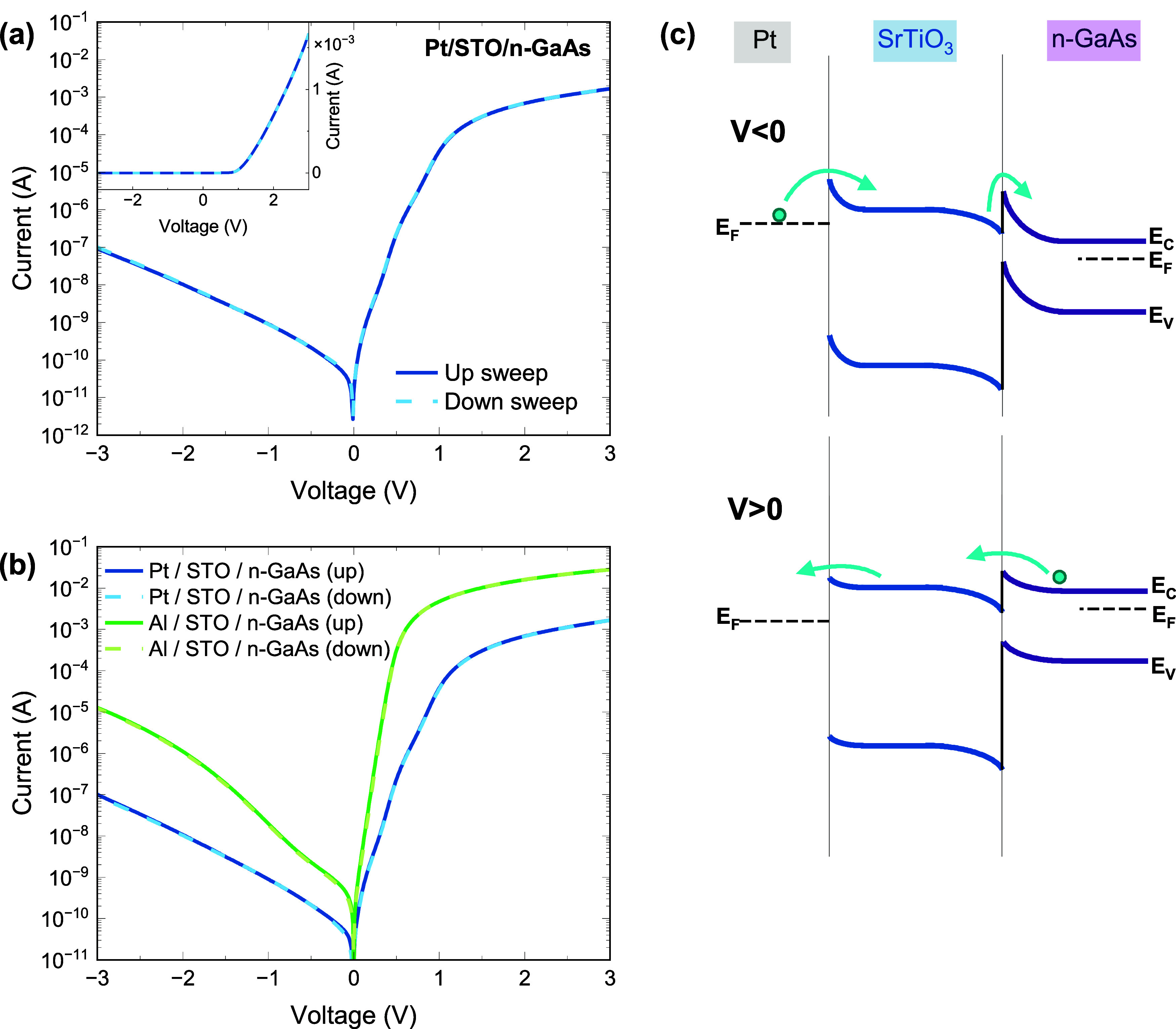
(a) Current–voltage curves of Pt/STO/n-GaAs structure in semilogarithmic and linear (inset) scales. (b) Comparison between Pt and Al top electrodes. (c) schematic energy band diagram of the Pt/STO/n-GaAs structure under positive and negative voltages. Note that the typical band bending in the GaAs is much wider than the thickness of the STO layer.

The transport behavior across the n-structure (STO/n-GaAs) resembles a mirror image of that of the p-structure. The vertical *I*–*V* characteristics (Figure 5a) exhibit opposite rectifying behavior, where high currents are measured under positive applied voltage and lower currents are measured under the reversed bias. The rectification observed in the isotype heterojunction results from the negative conduction band offset (CBO) at the STO/n-GaAs interface (Figure 3b). The CBO forms a potential barrier from the oxide side into n-GaAs; this limits the flow of electrons into the n-GaAs side when a negative voltage is applied. In addition, the transport of the electrons might be limited by a Schottky barrier at the Pt/STO interface at the metal electrode.^51^ In order to assess the role of the STO/GaAs interface in the total transport behavior across the structure, another set of heterostructures was prepared, where Al pads are used as top electrodes. The lower work function (WF) of Al compared to Pt^52,53^ is expected to significantly reduce the effect of the metal/oxide barrier^51^ on the electron behavior. The results, presented in Figure 5b, reveal that the strong rectifying character of the transport across the n-structure is preserved, indicating the dominant role of the STO/n-GaAs interface.

By contrast, in the p-structure, the use of Al has a much smaller effect on the *I*–*V* curve (Figure 4b). From the comparison in Figure 5b, we conclude that the Schottky barrier at the Pt/STO interface limits both the forward and the reverse currents and that the *I*–*V* characteristics of the Al/STO/n-GaAs structure provide a better probe of the transport across the STO/n-GaAs junction. The rectification ratio observed for this structure is 2 × 10^5^ at ±1 V, and an ideality factor of 1.3 is extracted from the low forward bias region (0.05–0.4 V). The STO/n-GaAs heterojunction resembles a Schottky junction, where the barrier height is expected to be approximately equal to the CBO and the measured reverse currents depend on electrons that cross from the STO conduction band to the GaAs. According to the thermionic emission (TE) model, the reverse saturation current is given by^48^, where *S* is the area of the junction, A* is the effective Richardson constant, and ϕ_B_ is the barrier height. When *I*_0_ is determined from the extrapolation of the forward current of the Al/STO/n-GaAs structure’s characteristics, and A* is calculated by taking the effective mass (m*) of GaAs to be 0.063*m*_0_,^48^ the TE model yields a ϕ_B_ of 0.68 eV. This value is in good agreement with the CBO value of 0.86 ± 0.15 eV (Figure 3), which is determined from XPS analysis. Although the determination of A* using m* = 0.063*m*_0_ is not necessarily accurate for the junction under consideration, the sensitivity of ϕ_B_ to the effective mass is relatively subtle. For example, for an m* value of 4.8*m*_0_, which is typical for STO,^54^ we extract a barrier height of 0.78 eV, which is closer to our spectroscopic determination. We note that the slight discrepancy between ϕ_B_ and CBO values could be related to the voltage drop across surface states at the STO/GaAs interface and to image-force barrier lowering. Furthermore, the barrier height in Schottky diodes is typically bias-dependent due to image-force barrier lowering, which agrees with the strong bias-dependency of the measured reverse currents (Figure 5b). The small deviation of the ideality factor from the ideal value of *n* = 1 suggests that the TE mechanism is not the only source of the measured currents. Tunneling of electrons through the barrier, by either field emission (FE) or thermionic field emission (TFE), is one plausible mechanism that could explain the ideality factor and the forward currents. It could also explain the bias-dependency of the reverse currents, together with the image-force barrier lowering effect.^55^

We note that under various conditions, STO and metal/STO junctions can exhibit a memristive behavior.^56−60^ To ensure the validity of our results, all of the *I*–*V* data presented here was acquired in a bidirectional scanning mode (Figures 4 and 5). The complete absence of hysteresis ensures that our results are not affected by memristive behavior despite the small degree of oxygen vacancies present in the films (Figure 2b). We ascribe this to the employment of e-beam evaporation for the metallization process, which circumvents most of the deposition surface damage which is common in sputtered contacts. We further ascribe the lack of memristive behavior to the lower degree of oxygen deficiency in our STO films, whereas in memristor devices oxygen deficiency is often introduced by design.

**Figure 6:**
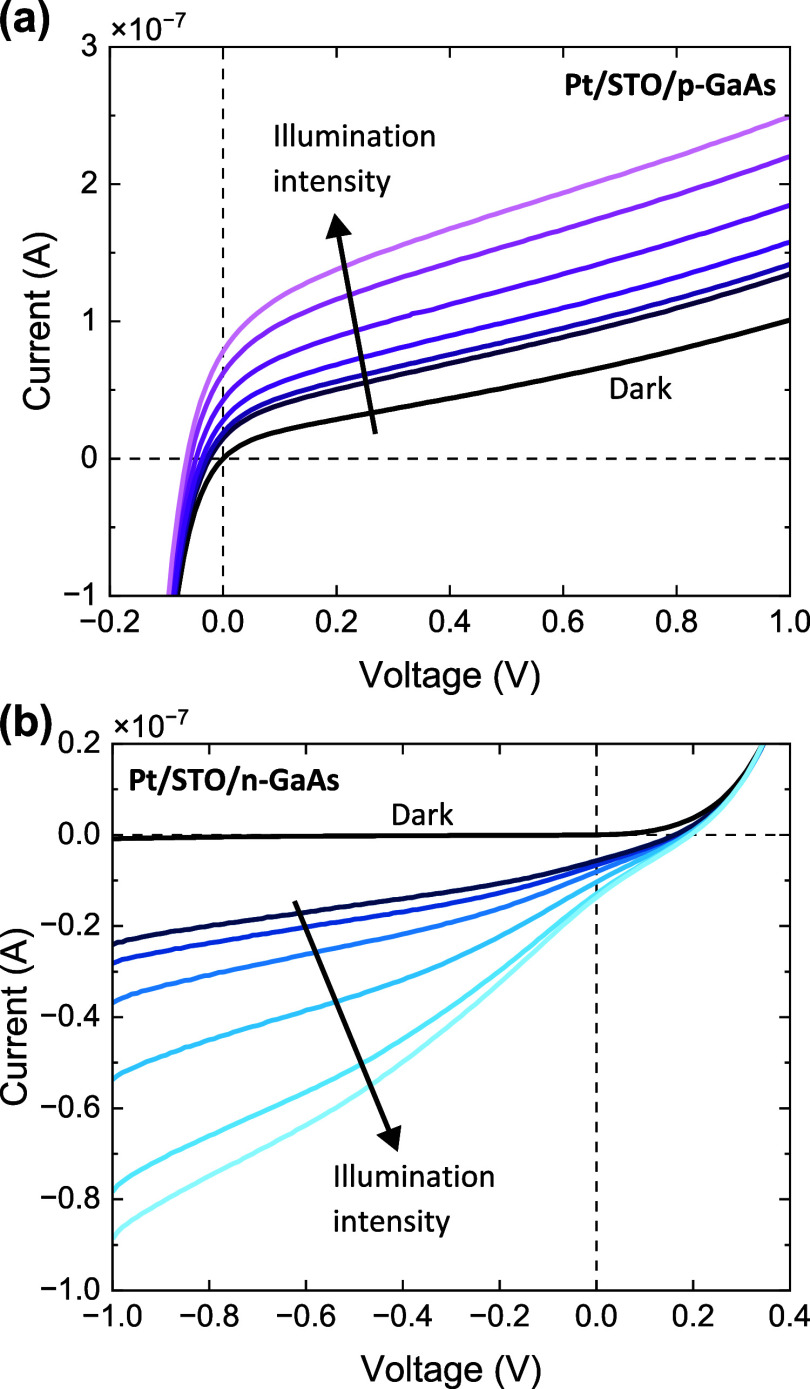
Current–voltage curves of (a) the Pt/STO/p-GaAs structure and (b) the Pt/STO/n-GaAs structure under varied and arbitrary illumination intensities.

The optical response of the heterostructures was estimated qualitatively (Figure 6) by monotonically and arbitrarily increasing the intensity of the microscope lamp in the probe station (150 W halogen lamp). External voltage was applied on Pt pads with an area of 1.1 × 10^–4^ cm^2^; the effect of illumination is likely in proximity to the pads’ edge. While rudimentary, this exploratory experiment yields several observations. First and foremost, a strong photoresponse is observed for both heterostructures, with a ∼×2.5 current increase for the p-structure at +1 V, and ∼×90 increase for the n-structure at −1 V. These indicate that the electro-optical functionality of the GaAs is not drastically degraded by our oxide epitaxy process, in agreement with the structural analysis (Figure 1). However, a quantitative analysis is required for a better assessment of this aspect.

For the p-structure (Figure 6a), the photoresponse occurs almost exclusively in positive voltages (“reverse bias” in the diode framework). This behavior is in good agreement with the band structure (Figure 4c), showing that under positive voltages, the p-GaAs depletion region is the largest. The source of the photocurrent is the separation of the photogenerated electron–hole pairs by the electric field in the depletion region.^48^ Both the field size and depletion region width increase as the positive voltage increases, showing excellent agreement with the results. Conversely, for the n-structure, the photoresponse is strong under negative voltages (Figure 6b), where the depletion region is the largest (Figure 5c). Altogether, the photoresponse results illustrate that STO/GaAs heterostructures maintain electro-optical functionality, the nature of which supports the band structure picture, in agreement with the XPS and *I*–*V* results (Figures 3–5). Further study is necessary to determine the wavelength and intensity dependence of the photoresponse.

## Conclusions

We studied the electrical properties of epitaxial SrTiO_3_/GaAs heterostructures through spectroscopic determination of the band alignment and analysis of transport behavior. We report a type II heterojunction with valence and conduction band offsets of 2.64 ± 0.15 and 0.86 ± 0.15 eV, respectively. These potential barriers dominate the charge carrier transport across the interfaces and are found to induce opposite rectifying behaviors across SrTiO_3_/p-GaAs and SrTiO_3_/n-GaAs heterojunctions. We further construct band structures of the two systems through which we outline a fundamental understanding of the electrical behavior across the SrTiO_3_/GaAs interfaces. An observed photoresponse in the reverse bias regions supports our transport analysis and indicates prospective electro-optical functionality. These results lay the foundations for implementation of oxide/GaAs interfaces in new devices and technologies.

## Supporting Information Available

XRD and AFM images of STO/n-GaAs and STO/p-GaAs structures, and the raw data used for plotting of the graphs (ZIP)
