# Supplementary material for: The Role of Interface Band Alignment in Epitaxial SrTiO3/GaAs Heterojunctions
Source: ACS Appl Electron Mater. 2024 Sep 16;6(10):7235–43. doi: 10.1021/acsaelm.4c01150 (PMC11500420; doi:10.1021/acsaelm.4c01150)
Supplement: Supplementary file 1 — el4c01150_si_001.zip [file el4c01150_si_001.zip › Supporting information_SrTO3-GaAs heterojunctions.pdf]

Supporting Information for

The Role of Interface Band Alignment in Epitaxial $SrTiO_3$/GaAs Heterojunctions

Shaked Caspi[1], Maria Baskin[1], Sergey Shay Shusterman[2,3], Di Zhang[4], Aiping Chen[4], Doron Cohen-Elias[2,3], Noam Sicron[2,3], Moti Katz[2,3], Eilam Yalon[1], Nini Pryds[5] and Lior Kornblum[1]*

[1]The Andrew & Erna Viterbi Dept. of Electrical and Computer Engineering, Technion – Israel Institute of Technology, Haifa 32000-03 – Israel

[2]The Israel Center for Advanced Photonics, 81800, Yavne, Israel

[3]Applied Physics Division, Solid State Physics Department, Soreq NRC, 81800, Yavne, Israel

[4]Center for Integrated Nanotechnologies (CINT) Los Alamos National Laboratory Los Alamos, NM 87545, USA

[5]Department of Energy Conversion and Storage, Technical University of Denmark (DTU), DK-2800 Kongens Lyngby, Denmark

*Corresponding Author: liork@technion.ac.il

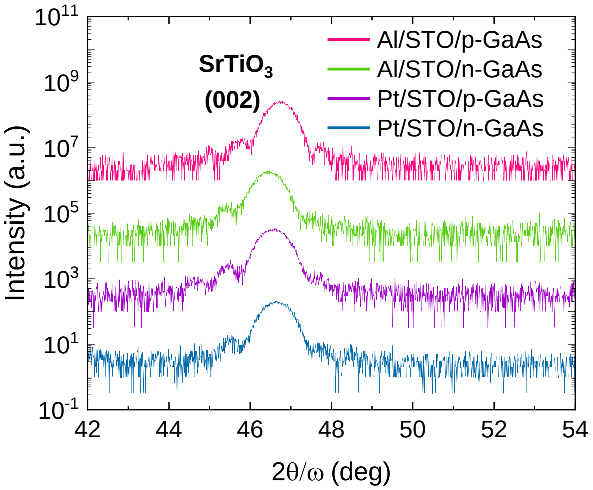

Figure S1 X-ray diffractions of (002) Bragg peak of $SrTiO_3$ of p-structures and n-structures with Pt and Al top electrodes.

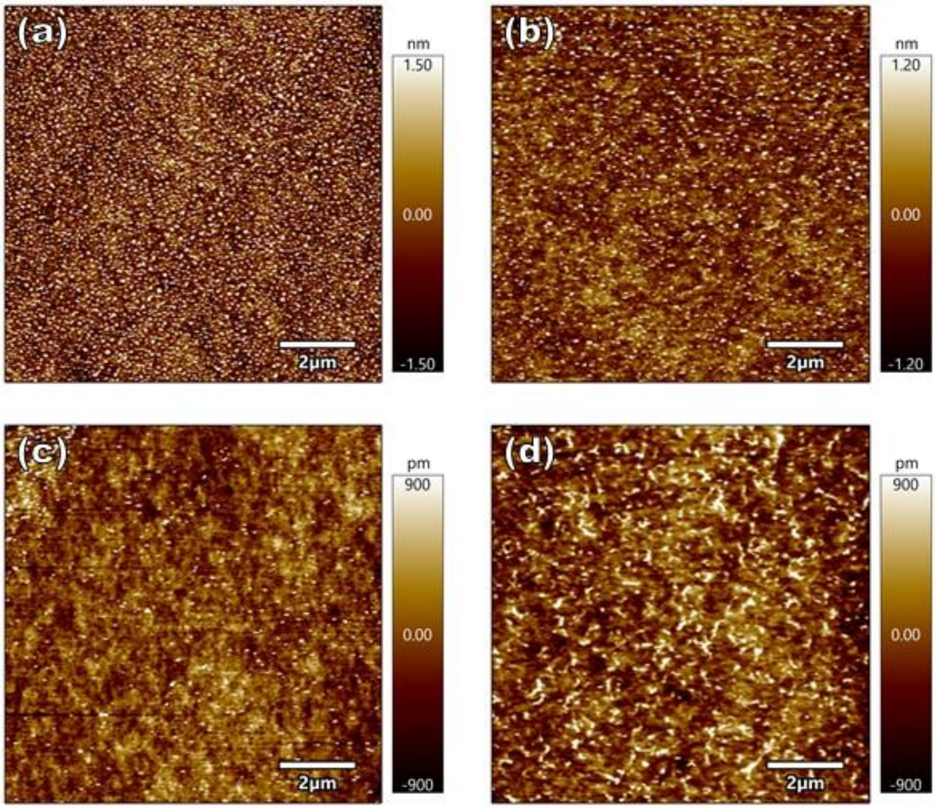

Figure S2 AFM images of $SrTiO_3$ surfaces in (a) Pt/STO/n-GaAs, (b) Al/STO/n-GaAs (c) Pt/STO/p-GaAs and (d) Al/STO/p-GaAs structures.

Table S1 Summary of root mean square (RMS) roughness calculated from AFM images.

**Structure**	**RMS roughness [pm]**
"Thick" STO/p-GaAs	430
Pt/STO/n-GaAs	900
Al/STO/n-GaAs	810
Pt/STO/p-GaAs	460
Al/STO/p-GaAs	440
